# Hydrothermal alteration and Cu–Ni–PGE mobilization in the charnockitic rocks of the footwall of the South Kawishiwi intrusion, Duluth Complex, USA

**DOI:** 10.1016/j.oregeorev.2014.11.010

**Published:** 2015-06

**Authors:** Zsolt Benkó, Aberra Mogessie, Ferenc Molnár, Kurt Krenn, Simon R. Poulson, Steven Hauck, Mark Severson, Greg B. Arehart

**Affiliations:** a Institute of Earth Sciences, University of Graz, Universitätsplatz 2, 8010 Graz, Austria; b Geological Survey of Finland, Betonimiehenkuja 4, 02151 Espoo, Finland; c Department of Geological Sciences & Engineering, University of Nevada — Reno, 1664 N. Virginia St., Reno, NV 89557-0138, USA; d Natural Resources Research Institute, University of Minnesota, 5013 Miller Trunk Highway, Duluth, MN 55811-1442, USA

**Keywords:** Duluth Complex, South Kawishiwi intrusion, Charnockite, Hydrothermal alteration, Fluid inclusions, Sulfur isotopes

## Abstract

In the Neoarchean (~ 2.7 Ga) contact metamorphosed charnockitic footwall of the Mesoproterosoic (1.1 Ga) South Kawishiwi intrusion of the Duluth Complex, the primary metamorphic mineral assemblage and Cu–Ni–PGE sulfide mineralization is overprinted by an actinolite + chlorite + cummingtonite + prehnite + pumpellyite + quartz + calcite hydrothermal mineral assemblage along 2–3 cm thick veins. In calcite, hosted by the hydrothermal alteration zones and in a single recrystallized quartz porphyroblast, four different fluid inclusion assemblages are documented; the composition of these fluid inclusions provide p–T conditions of the fluid flow, and helps to define the origin of the fluids and evaluate their role in the remobilization and reprecipitation of the primary metamorphic sulfide assemblage.

Pure CO_2_ fluid inclusions were found as early inclusions in recrystallized quartz porphyroblast. These inclusions may have been trapped during the recrystallization of the quartz during the contact metamorphism of the footwall charnockite in the footwall of the SKI. The estimated trapping pressure (1.6–2.0 kbar) and temperature (810–920 °C) conditions correspond to estimates based on felsic veins in the basal zones of the South Kawishiwi intrusion.

Fluid inclusion assemblages with CO_2_–H_2_O–NaCl and CH_4_–N_2_–H_2_O–NaCl compositions found in this study along healed microfractures in the recrystallized quartz porphyroblast establish the heterogeneous state of the fluids during entrapment. The estimated trapping pressure and temperature conditions (240–650 bar and 120–150 °C for CO_2_–H_2_O–NaCl inclusions and 315–360 bar and 145–165 °C for CH_4_–N_2_–H_2_O–NaCl inclusions) are significantly lower than the p–T conditions (> 700 °C and 1.6–2 kbar) during the contact metamorphism, indicating that this fluid flow might not be related to the cooling of the Duluth Complex and its contact aureole. The presence of chalcopyrite inclusions in these fluid inclusions and in the trails of these fluid inclusion assemblages confirms that at least on local scale these fluids played a role in base metal remobilization. No evidences have been observed for PGE remobilization and transport in the samples. The source of the carbonic phase in the carbonic assemblages (CO_2_; CH_4_) could be the graphite, present in the metasedimentary hornfelsed inclusions in the basal zones of the South Kawishiwi intrusion.

The hydrothermal veins in the charnockite can be characterized by an actinolite + cummingtonite + chlorite + prehnite + pumpellyite + calcite (I–II) + quartz mineral assemblage. Chlorite thermometry yields temperatures around 276–308 °C during the earliest phase of the fluid flow. In the late calcite (II) phase, high salinity (21.6–28.8 NaCl + CaCl_2_ equiv. wt.%), low temperature (90–160 °C), primary aqueous inclusions were found. Chalcopyrite (± sphalerite ± millerite), replacing and intersecting the early hydrothermal phases, are associated to the late calcite (II) phase. The composition of the formational fluids in the Canadian Shield is comparable with the composition of the studied fluid inclusions. This suggests that the composition of the fluids did not change in the past 2 Ga and base metal remobilization by formational fluids could have taken place any time after the formation of the South Kawishiwi intrusion.

Sulfur isotope studies carried out on the primary metamorphic (δ^34^S = 7.4–8.9‰) and the hydrothermal sulfide mineral assemblage (δ^34^S = 5.5–5.7‰) proves, that during the hydrothermal fluid flow the primary metamorphic ores were remobilized.

## Highlights

• Hydrothermal Cu–Ni mineralization is present in the footwall of the South Kawishiwi intrusion.
• S isotope values prove remobilization of the primary magmatic/metamorphic ores.
• Mixing carbonic–aqueous fluids (CO_2_ + H_2_O and CO_2_ + CH_4_ + N_2_) transported copper.
• High-salinity calcium-rich basinal brines remobilized copper and nickel.

## 1 Introduction

The world's largest Cu–Ni–PGE (platinum group elements) resources are hosted by large layered intrusions such as the Bushveld Complex, Noril'sk Camp, Sudbury Igneous Complex or the Duluth Complex (DC). The volumetrically important base and precious metal ores in these intrusions were produced by segregation, fractionation and settling of immiscible magmatic sulfide (Li and Ripley, 2011; Naldrett, 2010), but other models involving the significance of fluids and halogens also exist (Boudreau and McCallum, 1992). Devolatilization, desulfurization and/or partial assimilation of the country rocks is also a principal process of ore formation in mafic intrusions (Naldrett, 2010).

It has been presented recently that high grade, though small volume Cu–Ni and PGE mineralization may also occur in the footwall lithologies of layered intrusions (e.g., New Rambler deposit: McCallum et al., 1976; Nyman et al., 1990, Sudbury Igneous Complex: Molnár et al., 1997, 1999, 2001; Péntek et al., 2008, 2013; Tuba et al., 2010, 2013). Fluid inclusion and geochemical studies on hydrothermal alteration zones around mafic–ultramafic complexes reveal that fluids were produced by the contact metamorphism, partial melting of the footwall, or enter the system from external sources. These fluids played a significant role in the remobilization of primary magmatic sulfide and in the transport of metals in the footwall of these magmatic complexes.

Locally, high-grade mineralization was also reported in the metagranitoid footwall (WM-001 and other drill cores) of the South Kawishiwi intrusion (SKI) by Patelke (2003). No metal concentration data are available from the studied WM-002 drill core, but both the intrusion and the footwall part of the near-by WM-001 drill core was systematically analyzed (Patelke, 2003). The Cu content in the granitoid footwall is locally up to 1.91 wt.%, and the TPM is markedly high in the 338–383 m and in the 391–392 m depth interval with up to 827 ppb. Locally in the footwall Pd concentration is anomalously high, up to 860 ppb. Metal concentration data from the footwall, comparable with metal concentrations in the SKI underline the economic significance of mineralization in the footwall.

Hydrothermal processes and their roles in PGE remobilization in the troctolitic intrusions of the DC have been demonstrated by Mogessie et al. (1991), Ripley et al. (1993), Severson (1994) and Gál et al. (2011, 2013). Studies of Mogessie et al. (1991) conclude that Cu and PGEs were remobilized from the primary magmatic mineralization along fracture zones by C–O–H–S and Cl-rich fluids. Gál et al. (2011) described vein-type, hydrothermal Cu-mineralization associated with actinolite–chlorite–prehnite–pumpelleyite–calcite alteration assemblage in the hanging-wall of the SKI at the Filson Creek deposit. In this work remobilization of Pd from the primary magmatic Cu–Ni–PGE mineralization to an unknown location was also demonstrated and it was shown that the late serpentinization of the intrusion resulted in only very local scale remobilization of base metals and PGEs. Hydrothermal remobilization of primary ores has also been demonstrated in the Babbitt Cu–Ni deposit (Bathtub intrusion) by Ripley et al. (1993). Based on stable isotope studies (δD and δ^18^O for H_2_O) they proved that fluids both from magmatic and metasedimentary footwall sources were involved in the Pt–Pd redistribution. These studies have the following economic significance: 1.) fluid circulation in the primary mineralized zones of the intrusion may have resulted in depletion of the ores in PGEs, and 2.) there is a high probability of the formation of secondary hydrothermal Cu-mineralization also far from the basal mineralized zones, in the hanging wall or in the footwall units of the intrusion.

The aim of this paper is to present the textural, mineralogical and geochemical characteristics of the hydrothermal alteration zones in the charnockitic footwall of the SKI and to characterize the hydrothermal fluids based on fluid inclusion studies. Fluid inclusion studies have not only been carried out on footwall samples, but also on non-metamorphosed and unaltered granitoid samples far from the contact aureole, in order to distinguish regional and local fluid flow events.

## 2 Geologic setting

### 2.1 Regional geology of the Duluth Complex

**Fig. 1:**
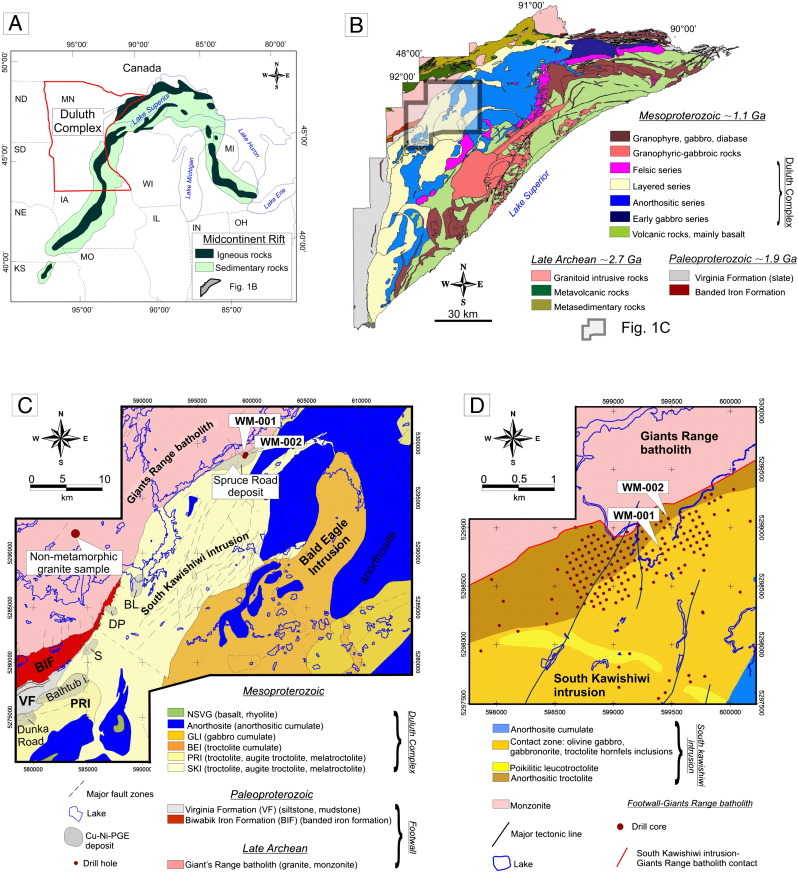
(A) The Midcontinent Rift system in North -America (modified after Ojakangas et al., 2001). (B) Geologic map of the DC. The South Kawishiwi intrusion, studied in this work is highlighted by a black square. (C) Geology of the SKI and its surroundings. Abbreviations: BL — Birch Lake Deposit, DP — Dunka Pit Deposit, S — Serpentine Deposit, Partridge River intrusion. (D) Detailed geologic map of the Spruce Road deposit.

The DC and associated intrusions in northeastern Minnesota are part of the Mesoproterozoic (1.1 Ga) Midcontinent Rift (MCR; Fig. 1A) which is exposed in the Lake Superior region. Intrusive and effusive rocks with the MCR cover ca. 5700 km^2^ arcuate area in northeastern Minnesota (Fig. 1B). The DC is defined as a continuous mass of mafic to felsic plutonic rocks that intruded between the Neoarchean to Early Proterozoic footwall and the co-magmatic rift related volcanic rocks of the hanging wall (Fig. 1B; Miller and Severson, 2002).

Miller and Severson (2002) distinguished four general rock series within the nearly continuous mass of intrusive rocks forming the DC, namely the Felsic Series, the Early Gabbro Series, the Anorthositic Series and the Layered Series. The Cu–Ni–PGE sulfide mineralization is hosted by three of the Layered series intrusions that were emplaced during the main stage of the rift related magmatism (Miller and Severson, 2002). The Cu–Ni–PGE laden mineralized intrusions are from NE to SW the South Kawishiwi intrusion, the Bathtub intrusion and the Partridge River intrusion (Fig. 1C).

The DC is in its current position tilted by 15–20° to SE, and therefore the immediate footwall units of the SKI, Bathtub intrusion and Partridge River intrusion crop out along the north-eastern perimeter of the DC.

### 2.2 Geology of the Duluth Complex footwall

Footwall rocks adjacent to and beneath the DC include Neoarchean intrusive, sedimentary and magmatic rocks and Paleoproterozoic sedimentary rocks of the Animikie Basin. Neoarchean granitoid rocks form the direct footwall with mineralized layered intrusions only the northeastern segment of the SKI. Granitic, granodioritic, monzonitic and tonalitic rocks comprise most of the 2.7 Ga Giants Range batholith (GRB), which is intrusive into the supracrustal rocks of the Wawa–Abitibi subprovince — the southernmost granite–greenstone belt of the Superior Province (Boerboom and Zartman, 1993).

Except for a short (~ 25 km in strike length) segment along the SKI, the footwall of the DC is the Early Proterozoic Biwabik Iron Formation and the graphite- and pyrite-bearing Virginia Formation. Multiple intrusions of cumulate textured anorthositic and troctolitic rocks produced high-temperature amphibole–pyroxene hornfels facies contact metamorphism, devolatilization and locally partial melting of the footwall lithologies (Sawyer, 2002; Sims and Viswanathan, 1972).

### 2.3 Cu–Ni–PGE sulfide mineralization in the basal zones of the layered intrusions of the Duluth Complex

The dominantly disseminated Cu–Ni–PGE sulfide mineralization of the SKI, Partridge River intrusion and Bathtub intrusions (Fig. 1C) is confined to the lower 100–300 m of the intrusions. The sulfide ore mineralization collectively constitutes over 4.4 billion tons of material averaging 0.66% Cu and 0.20% Ni at a 0.5% Cu cut-off (Listerud and Meineke, 1977) and is dominated by chalcopyrite + cubanite + pyrrhotite + pentlandite ± bornite. Locally along the lower contact of the intrusions volumetrically small, massive and semi-massive sulfide pods may also occur that are dominated by pyrrhotite and pentlandite (Severson and Hauck, 2008). Differences in sulfide assemblages and textures can be explained partly by magmatic processes and partly by heterogeneous footwall–intrusion interactions (Hauck et al., 1997; Mogessie and Stumpfl, 1992; Mogessie et al., 1991; Pasteris et al., 1995; Peterson, 2010; Ripley et al., 1993; Severson, 1994).

The emplacements of the intrusions (SKI, Bathtub intrusion, Partridge River intrusion) resulted in intense mineralogical, textural and geochemical modifications of the footwall. According to the studies of Ripley (1981), Andrews and Ripley (1989), Ripley and Al-Jassar (1987) and Mogessie and Stumpfl (1992), the sulfur δ^34^S values range from 0 to 26‰ and the sulfur in the basal zones of the Partridge River intrusion and the Bathtub intrusion is derived from the metasedimentary footwall. Ripley (1981) speculated early on that pyrite in the sulfide-bearing black slates of the Virginia Formation had become desulfurized to form pyrrhotite and SO_2_ or H_2_S. In addition — due to the fluid–rock interaction — the organic component (graphite) in the metasedimentary Virginia Formation liberated in the form of CO_2_ and CH_4_ during the contact metamorphism by the DC (Pasteris et al., 1995). The SO_2_ and H_2_S, as well as, felsic partial melts (Gál et al., 2013) migrated upward and triggered rapid sulfur saturation in the basal zones of the intrusions (Thériault et al., 2000). Upward migration of sulfur from the footwall was proven by sulfur isotope studies.

The late-stage, low-temperature (< 350 °C) hydrothermal fluid circulation resulted in local formation of actinolite + prehnite + pumpellyite + chlorite + calcite veins and alteration zones in the SKI and in the Partridge River intrusion (Gál et al, 2011; Mogessie et al., 1991). These fluids may also have remobilized and precipitated Cu, Ni and PGE-baring sulfide minerals along some tectonic zones of the troctolite and in the upper anorthositic sequences (Gál et al., 2011, 2013; Mogessie and Saini-Eidukat, 1992; Mogessie and Stumpfl, 1992; Mogessie et al., 1991; Ripley et al., 1993).

There are only a few studies dealing with characterization of hydrothermal fluids affecting DC mineralized zones by fluid inclusion studies. Pasteris et al. (1995) reported various fluid inclusion assemblages from the southern part of the DC with various temperatures (200–700 °C) and compositions (CO_2_ ± CH_4_ ± N_2_ and NaCl + CaCl_2_ + KCl) and with highly variable salinities (from 0 to 48 wt.% NaCl + CaCl_2_) and emphasized the potential of brine–gas and brine–brine immiscibility in modification of primary distribution of metals. Gál et al. (2013) investigated coexisting melt and CO_2_ (± CH_4_ ± N_2_) inclusions in local felsic segregations and cross-cutting felsic dikes presumably originated from partial melting of footwall in the SKI and suggested that the CO_2_-rich deuteric fluid phase originated from the mafic magma.

### 2.4 Geology and mineralization in the Giant Range batholith beneath the South Kawishiwi intrusion in the Spruce Road deposit area

Granitoid rocks of the GRB beneath the SKI belong to the Farm Lake Facies, which is a porphyritic hornblende- or biotite-bearing monzodiorite that consists of plagioclase + K-feldspar + quartz + biotite + hornblende (Boerboom and Zartman, 1993; Green, 1970; Fig. 1D). Based on the petrographic observations of Green (1970) and Sims and Viswanathan (1972) mineral assemblages in the contact aureole (hornblende + hyperstene + biotite + magnetite) of the DC are characteristic of amphibolite, hornblende–hornfels, and pyroxene hornfels facies. According to the estimates of Turner (1968) the rocks recrystallized in the temperature range of 600–675 °C and under pressures of roughly 1.5–2.5 kbar. Perry and Bonnichsen (1966) and Bonnichsen (1969) calculated 700–750 °C as a maximum temperature in the contact metamorphic zone. More recently, Sawyer (2002) gave a detailed description on the deformation and partial melting textures of a single drill core (WM-001) and concluded that the temperatures in the contact aureole could be as high as 800 °C in the pressure range of 1 to 3 kbar.

**Fig. 2:**
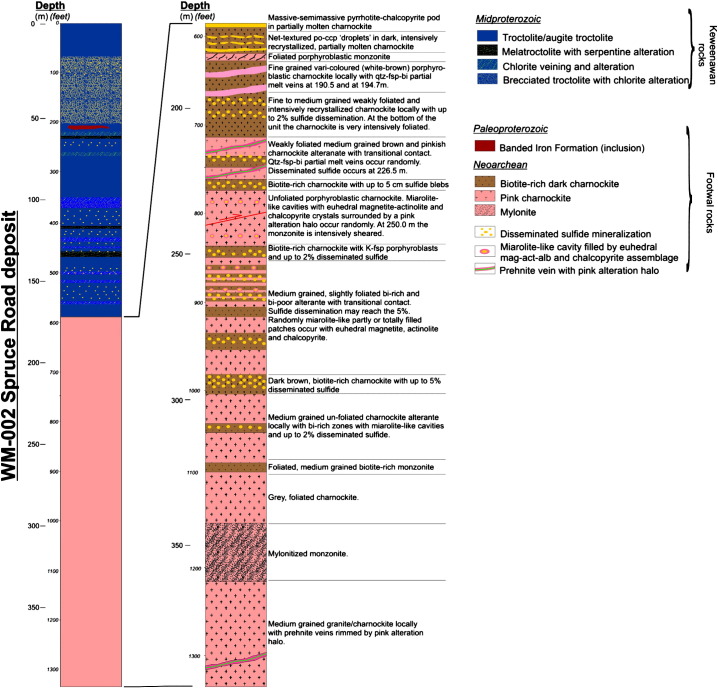
Log of the WM-002 drill core.

**Fig. 3:**
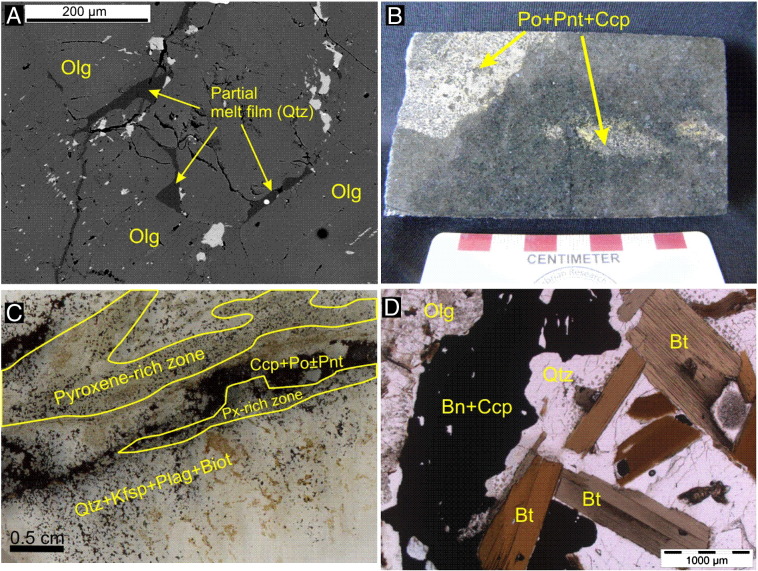
Evidences of partial melting and the relationship of partial melting and magmatic mineralization in the charnockitic footwall. (A) Cuspate formed partial melt film that crystallized to quartz (Qtz) between oligoclase porphyroblasts. Backscattered electron image. (B) Drop-like, semi-massive, net textured sulfide blebs composed of pyrrhotite (Po) + pentlandite (Pnt) + chalcopyrite (Ccp). (C) Pyroxene-rich veins. The green minerals in the vein are ortho-, and clinopyroxene. The vein is surrounded by a 2–3 cm thick halo that is composed of subhedral fine grained quartz + K-feldspar + plagioclase + biotite. In the axis of the vein chalcopyrite + pyrrotite ± pentlandite occurs. Scanned thin section. (D) Partial melt patch composed of quartz and biotite. In the partial melt patch bornite (Bn) and chalcopyrite (Ccp) occurs in form of granopyhric intergrowth. Parallel-polarized light image.

According to the recent studies of Benkó et al. (2013) carried out on the WM-001 and WM-002 drill cores (Figs. 1D and 2), in the contact aureole, the monzodiorite is transformed into a charnockite due to orthopyroxene, and clinopyroxene replacing biotite and hornblende (Green, 1970; Sawyer, 2002). Several evidences of partial melting (e.g. partial melt films; Fig. 3A) are presented in Sawyer (2002). The temperature in the proximal (0–10 m) contact zones may have been up to 900 °C (Benkó et al., 2013), and this resulted in intense partial melting and percolation of dense Fe-rich sulfide melt droplets (Fig. 3B) into the partially molten charnockite from the basal mineralized zone of the intrusion (Benkó et al., 2013; Fig. 3B). This mineralization is dominated by pyrrhotite + pentlandite + chalcopyrite. Towards the deeper zones (10–50 m) below the contact a Cu-rich sulfide assemblage occurs along pyroxene-rich veins (dominated by chalcopyrite + pyrrhotite and surrounded by a plagioclase + K-feldspar + quartz + biotite assemblage; Fig. 3C). In the distal zones (50–100 m from the contact) a more evolved assemblage occurs characterized by bornite + chalcopyrite and platinum group minerals (PGMs) associated to biotite + quartz partial melt patches (Fig. 3D).

No metal concentration data are available from the studied WM-002 drill core, but both the intrusion and the footwall part of the near-by WM-001 drill core was systematically analyzed (Patelke, 2003). Metal content in the intrusion part of the WM-001 drill core increases towards the footwall; the total Cu content is up to 0.91 wt.% and the Ni is 0.31 wt.% in the basal mineralized zone. The total precious metal concentration (TPM = Pt + Pd + Au) is up to 581 ppb in some zones of the BMZ. The Cu content in the charnockitic footwall is locally up to 1.91 wt.%, but the maximum Ni content is significantly lower (0.15 wt.%) than in the basal mineralized zone of the intrusion. The TPM is markedly high in the 338–383 m and in the 391–392 m depth interval with up to 827 ppb. Locally in the footwall Pd concentration is anomalously high, up to 860 ppm.

## 3 Sampling and methodology

Samples were collected from the drill core library of the Minnesota Department of Natural Resources in Hibbing. Two drill cores the WM-001 and WM-002, acquired by Wallbridge Mining from the Spruce Road area (Figs. 1 C and D) were systematically re-logged and sampled. Both drill cores traverse more than 100 m of the charnockitic monzodiorite of the GRB beneath the SKI contact. Three samples were collected from a road cut south from Ely, 11 km from the contact aureole of the DC.

Electron microprobe analysis on polished thin sections was performed on a JEOL 6310 SEM, equipped with a Link ISIS energy dispersive system and a Micro-Spec wavelength dispersive system at the University of Graz. Accelerating voltage was 15 kV, the probe current 5 nA for silicates and oxides. Detection limits are 0.1–0.2 wt.% for EDS and 0.03 wt.% for WDS analysis.

Sulfur isotope analyses were carried out in the isotope lab of the Department of Geological Sciences and Engineering, University of Nevada, Reno. Analyses were performed using a Eurovector EA 3000 elemental analyzer interfaced to a Micromass IsoPrime stable isotope ratio mass spectrometer, after the methods of Giesemann et al. (1994) and Grassineau et al. (2001). V_2_O_5_ was added to the samples as a combustion aid. δ^34^S results are reported in units of ‰ vs. VCDT. An uncertainty of ± 0.2‰ is recommended.

Fluid inclusions have been investigated in oriented doubly-polished thick sections (thickness ca. 0.15 mm). Measurements of phase transition temperatures in fluid inclusions were performed using a LINKAM THSMG600 heating and freezing stage with an operating range from − 196 °C to + 600 °C (Shepherd et al., 1985), equipped with an OLYMPUS 80 × ULWD objective. Temperature measurements are reproducible to within 0.2 °C at a heating rate of 0.1 °C/min. Raman spectroscopy was performed with a Jobin-Yvon LabRam-HR 800 Raman micro-spectrometer at the Institute of Earth Sciences, University of Graz, Austria by using a He–Ne laser with 633 nm excitation line and 5.9 mW at the sample.

For calculation of bulk fluid inclusion densities and molalities of CO_2_, CH_4_ and N_2_ bearing inclusions from microthermometric data (homogenization temperatures, clathrate melting temperatures, eutectic temperatures, ice melting temperatures) the module BULK of the Software Package Fluids v.1 was used (Bakker, 2003). Salinities in these inclusions were calculated using the equations of state of Duan et al. (1996). X_CH_4__/X_N_2__ molar ratios were determined from the areas of the representative peaks of the components on the Raman spectra using the method of Burke (2001). Isochores for CH_4_ + N_2_ and CO_2_ bearing inclusions were calculated by the Program Package FlinCor (Brown, 1989) using the equation of state of Kerrick and Jacobs (1981).

The salinities and densities of the aqueous fluid inclusions were calculated using the experimental equation of state (Bodnar, 1993; Oakes et al., 1990). Isochores were calculated using the equations after Zhang and Frantz (1987).

## 4 Results

### 4.1 Petrography

#### 4.1.1 Alteration assemblages

Two distinct assemblages of hydrothermal alteration minerals are found to replace the original igneous and contact metamorphic minerals in the charnockite.

**Fig. 4:**
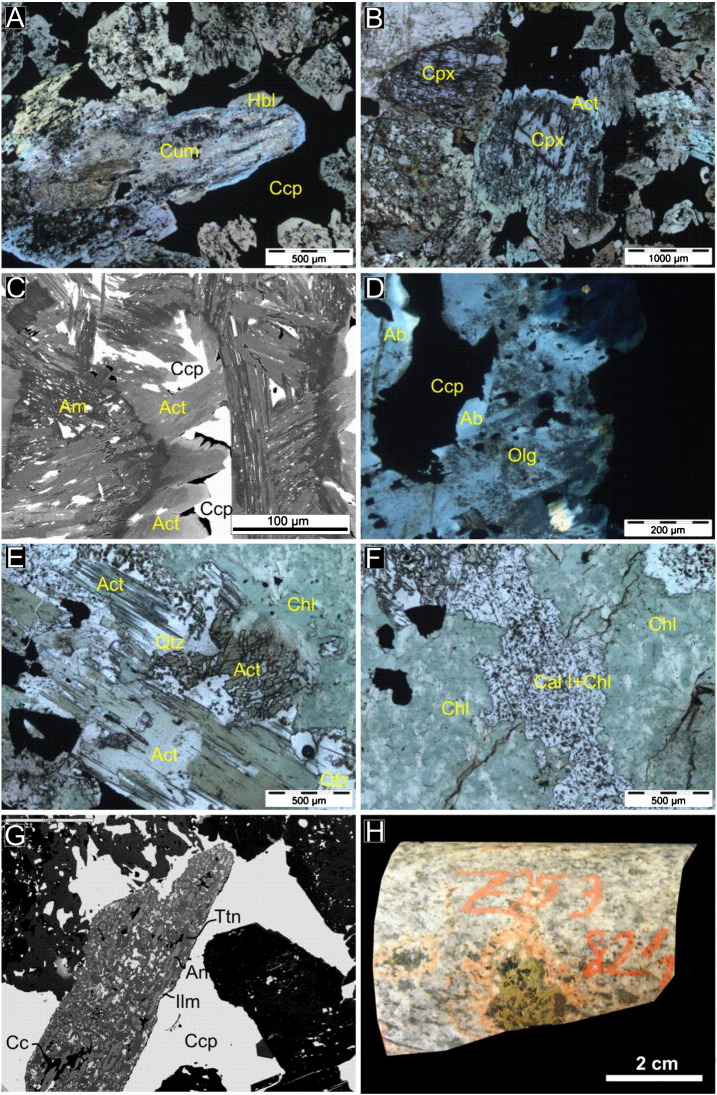
(A) Orthopyroxene is altered to cummingtonite (Cum). The altered grain grades towards the rim to hornblende (Hbl). Parallel-polarized light image. (B) Clinopyroxene (Cpx) with syntaxial overgrowth of actinolite (Act). Parallel-polarized light image. (C) Zoned, acicular actinolite crystals. Needle-like amphibole (Am) replaces chalcopyrite (Ccp). Corroded chalcopyrite grains occur along cleavage planes of the amphibole. Backscattered electron image. (D) Granoblastic oligoclase (Olg; inclusion-rich) is overgrown by albite (Ab; clear, gray). Cross-polarized light image. (E) Actinolite is replaced by quartz (Qtz) and chlorite (Chl). Quartz is intergrown with chlorite, indicating their simultaneous crystallization. Parallel-polarized light image. (F) Calcite intergrown with chlorite. Parallel-polarized light image. (G) Titanite (Ttn) replaced by anatase (An) + calcite I (Cc) + ilmenite (Ilm). Backscattered electron image. (H) The chlorite–calcite II–quartz–prehnite–pumpellyite alteration zones are surrounded by a 1–2 mm pink alteration halo.

The orthopyroxene in the charnockite that evidently formed during pyroxene hornfels metamorphism is altered along grain boundaries and along cleavage planes to a fine, fibrous mass of cummingtonite. The pseudomorphed orthopyroxene has a syntaxial overgrowth of a green-pale pleochroic amphibole (Fig. 4A). Metamorphic clinopyroxene (compositionally augite) has also been altered along cleavage planes and along grain boundaries to actinolite. Syntaxial overgrowth of clinopyroxene by actinolite has been documented in rare cases (Fig. 4B). Under transmitted light, a distinct growth zonation of individual amphibole crystals, with darker rims and pale cores, has been detected that implies decreasing Mg/(Mg + Fe) values towards the rims. Corroded remnants of chalcopyrite occur as inclusions or between individual actinolite crystals (Fig. 4C). Rock forming oligoclase of the charnockite, is locally replaced by albite (Fig. 4D).

**Fig. 5:**
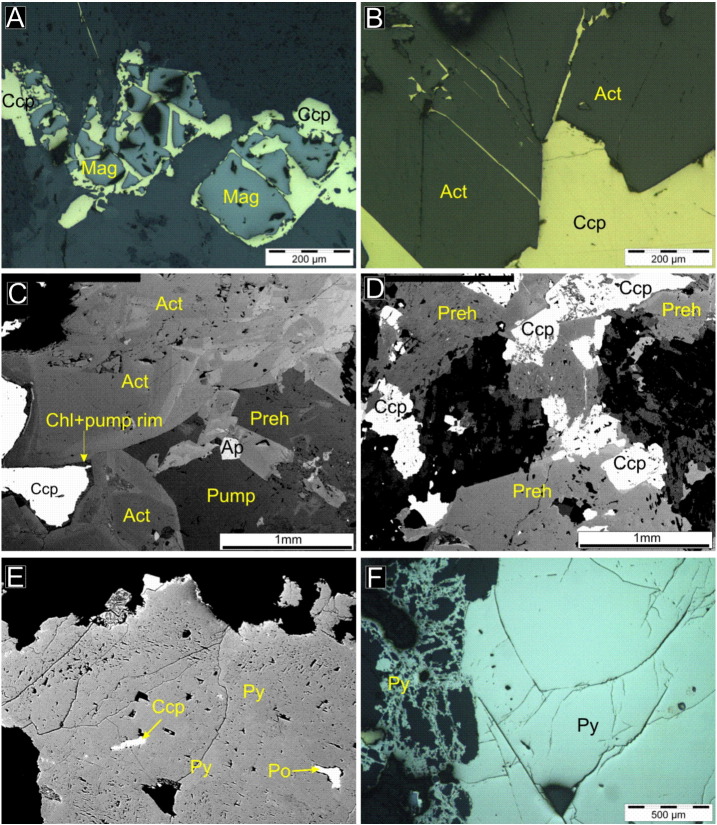
(A) Magnetite (Mag) is corroded and replaced by chalcopyrite along grain boundaries and cleavage planes. Reflected, parallel polarized light image. (B) Chalcopyrite invades along cleavage planes of actinolite. Reflected, parallel polarized light image. (C) Chalcopyrite surrounded by chlorite and prehnite halo between actinolite crystals. Backscattered electron image. (D) Prehnite intergrown with chalcopyrite. Backscattered electron image. (E) Corroded chalcopyrite and pyrrhotite crystals in fenestral textured pyrite. (F) Granular and skeletal textured pyrite intergrown with chlorite. Reflected, parallel polarized light image.

A second phase of alteration is characterized by the chlorite–quartz–calcite–prehnite–pumpellyite mineral assemblage in vugs up to 5 cm and in 2–3 cm thick veins. Fine-grained (50–100 μm) chlorite replaces rock-forming metamorphosed oligoclase of the parent charnockite host and actinolite that formed from the alteration assemblage affecting metamorphic pyroxene (Fig. 4E). That chlorite replaces actinolite after pyroxene implies that this assemblage is younger than the actinolite–cummingtonite assemblage. Calcite has two occurrences, calcite I and II. Calcite I also replace actinolite and fills open vugs along with prehnite and pumellyite. Anhedral calcite I and quartz with chlorite are often intergrown indicating simultaneous growth from hydrothermal solutions (Fig. 4F). Prehnite and pumpellyite form up to 1 mm fan-shaped crystal masses replacing actinolite and feldspar. Titanite that formed in the first phase in association with amphibole is altered in the second phase to fine-grained (10–50 μm) anatase + calcite + ilmenite (Fig. 4G). Calcite II occurs along veins is clear and rich in fluid inclusions. It is partly intergrown with prehnite and pumpellyite or replaces all precursor hydrothermal mineral phases (actinolite, chlorite, prehnite, pumpelleyite). The hydrothermally altered parts of the charnockite (veins and vugs) are surrounded by a 1–2 mm alteration halo that occurs as a pink margin along these veins and vugs on the hand specimen scale (Fig. 4H). In this alteration selvage, oligoclase is sericitized and albitized. Sericite occurs in the plagioclase as 5–25 μm needles. Magnetite is corroded along grain boundaries and fractures and is overgrown by a second generation of chalcopyrite (Fig. 5A). Chalcopyrite occurs also along cleavages and fractures of actinolite (Fig. 5B). A second generation of chalcopyrite is rimmed by chlorite and prehnite (Fig. 5C–D). Galena, sphalerite and millerite occur as inclusions in chalcopyrite.

Where the fluids of the second hydrothermal assemblage affected the sulfide assemblage that filtrated during the contact metamorphism in the partially molten charnockite, the pyrrhotite and the chalcopyrite are intensively corroded and occur as inclusions in the pyrite (Fig. 5E). Pyrite occurs in three textural forms. Where it corrodes the primary pyrrhotite and chalcopyrite, it has a characteristic massive or fenestrated texture (Fig. 5E). Along millimeter scale chlorite veins that cut the rock forming silicates (oligoclase or pyroxene), pyrite forms euhedral skeletal crystals (Fig. 5F). Pyrite locally also forms octahedral or cubic crystals. No direct petrographic evidence for replacement of pentlandite by millerite has been found, but in the second hydrothermal alteration assemblage occurrences, the Ni-bearing sulfide phase is millerite. It is massive, granular, locally subhedral.

#### 4.1.2 Quartz porphyroblast

**Fig. 6:**
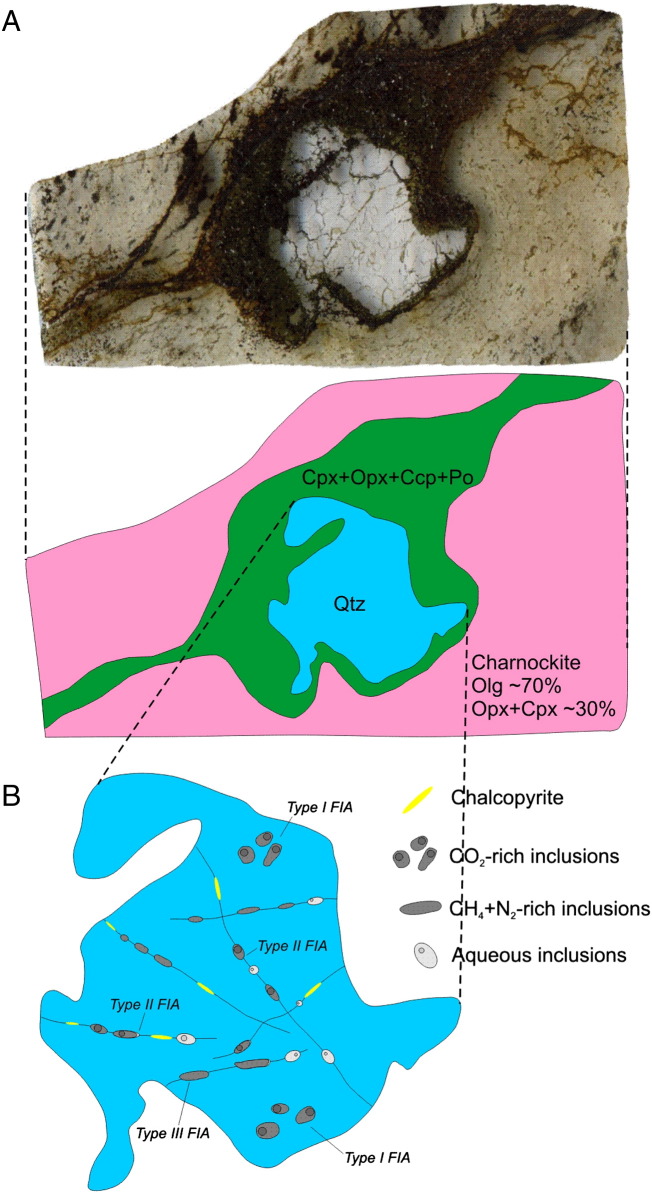
(A) Quartz porphyroblast mantled by pyroxene + pyrrhotite + chalcopyrite. (B) Fluid inclusion assemblages in the quartz porphyroblast.

In the WM-002 drill core, beside the calcite crystals in the hydrothermal veins, only one relict quartz porphyroblast, 10 m beneath the intrusion–footwall contact was found suitable for fluid inclusion studies (Fig. 6).

The porphyroblast consists of a polygonal aggregate of quartz crystals, without internal stress features (undulose extinction, etc.). The crystals have straight or slightly rounded grain boundaries and a 120° triple junction between grains, indicating equilibrium recrystallization.

The mechanism of formation of the porphyroblast is enigmatic, since the modal proportion of the quartz in the host partially molten charnockite is low (< 1%). Quartz occurs only as cuspate formed crystals between feldspar porphyroblasts (Sawyer, 2002) or in higher proportions in association with subhedral, fine grained (50–200 μm) K-feldspar and plagioclase in patches that occur locally and are interpreted as crystallized partial melts.

The porphyroblast is mantled by a 1 mm thick halo composed of clinopyroxene, orthopyroxene, and chalcopyrite + pyrrhotite with minor galena and sphalerite as accessories (Fig. 6A). The quartz is in direct contact with chalcopyrite. The quartz porphyroblast consists of several fractures with random orientation that terminate in the grain boundary of the quartz grain. These fractures are partly filled by chalcopyrite. Occurrence of various fluid inclusion assemblages is also common along these fractures (Fig. 6B; fluid inclusion petrography, see below).

However, only one single quartz porphyroblast was found in the drill core, this single crystal can be representative since it is mantled by ortho- and clinopyroxene that formed during the peak metamorphism. Therefore, the quartz has probably also recrystallized during the metamorphism. On the other hand this is the only mineral in the drill cores that may have preserved fluids that were present during the peak of the contact metamorphism.

### 4.2 Chlorite chemistry

**Fig. 7:**
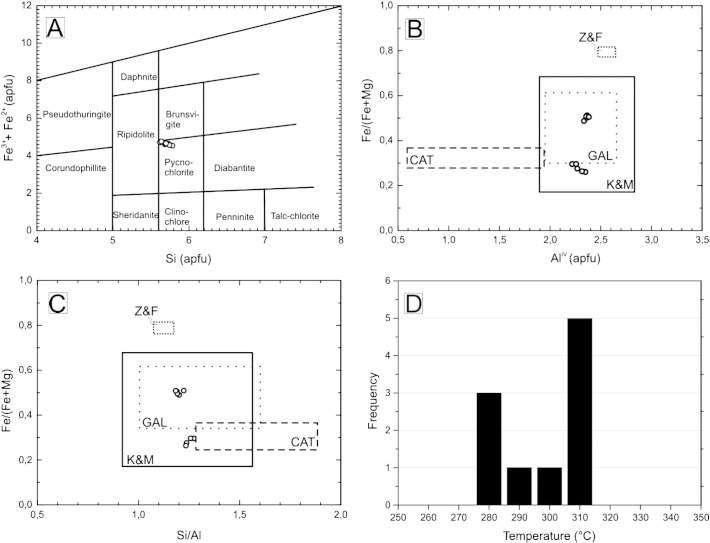
(A) Classification of chlorites based on the diagram of Hey (1954). (B–C) Comparison of chlorite compositions from the charnockitic footwall of the SKI with those used for empirical temperature estimation of Cathelineau (1988), Kranidiotis and MacLean (1987), and Zang and Fyfe (1995). Compositional fields of the Anorthositic series of the SKI of the DC (Gál et al., 2011) are plotted for reference. (D) Histogram of crystallization temperatures of chlorites calculated by the empirical method of Kranidiotis and MacLean (1987).

Electron microprobe analyses and temperature of crystallization are listed in Table 1. Analyses were recalculated on the basis of 28 oxygen atoms per formula unit. Fig. 7A shows the classification of chlorites based on the criteria of Hey (1954). Chlorite compositions, in the studied samples, fall in the pycnochlorite field with Si values ranging from 5.62 to 5.78 [atoms per formula unit (*apfu*)] and total Fe [= Fe^2 +^ + Fe^3 +^ (*apfu*)] values ranging from 2.53 to 4.84 (*apfu*). The Fe/(Fe + Mg) ratio varies from 0.26 to 0.5; the Fe^3 +^ value is very low, characterizing the chlorites to be Fe-rich and unoxidized. The Si/Al ratio shows very little variability, ranging from 1.18 to 1.27 and the Al in the tetrahedral site varies between 2.53 and 2.84 ions per formula unit (Fig. 7B and D).

### 4.3 Sulfur isotope data

**Fig. 8:**
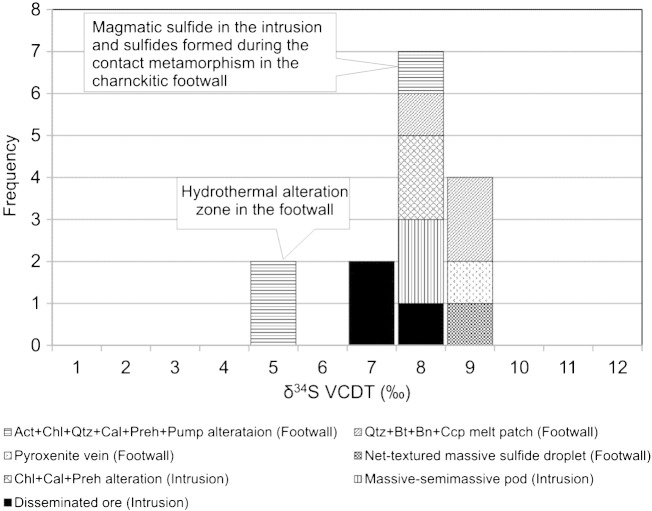
Histogram showing distribution of δ^34^S values for sulfides in troctolitic, charnockitic and in their hydrothermally altered equivalents at the Spruce Road deposit of the SKI.

In order to compare isotopic variations during magmatic, metamorphic and hydrothermal processes, the following mineralization types were sampled in the drill cores in this study: (1) disseminated type chalcopyrite–cubanite–pentlandite ore in the basal mineralized zone, (2) semi-massive pyrrhotite–pentlandite–chalcopyrite dominated mineralized basal zone of the intrusion, (3) chalcopyrite-rich chlorite–calcite–prehnite veins and patches in the troctolite, (4) pyrrhotite–pentlandite–chalcopyrite dominated sulfide droplets that percolated into the proximal zones of the footwall during peak metamorphism, (5) pyroxenite-veins with chalcopyrite and pentlandite, (6) bornite–chalcopyrite composite grains associated with quartz–biotite melt patches and (7) chalcopyrite-rich ore associated to chlorite–quartz–calcite–prehnite–pumpellyite alteration assemblage. Results of sulfur isotope analyses are listed in Table 2 and plotted in Fig. 8. Except for two analyses, all δ^34^S values irrespective of host rock, mineral assemblage or texture fall in a narrow range, between + 7.4 and + 8.9 with an average value of + 8.0‰. The two samples having considerably lower δ^34^S values (+ 5.5 and + 5.7‰) represent the samples from the footwall with actinolite–chlorite–quartz–calcite–prehnite–pumpellyite alteration.

### 4.4 Fluid inclusion studies

#### 4.4.1 Fluid inclusion petrography

Four different fluid inclusion assemblages (FIA) have been distinguished on the basis of the host mineral, petrographic location and the microthermometric behavior. Petrographic and microthermometric properties of the different FIA and the calculated salinities are summarized in Table 3.

**Fig. 9:**
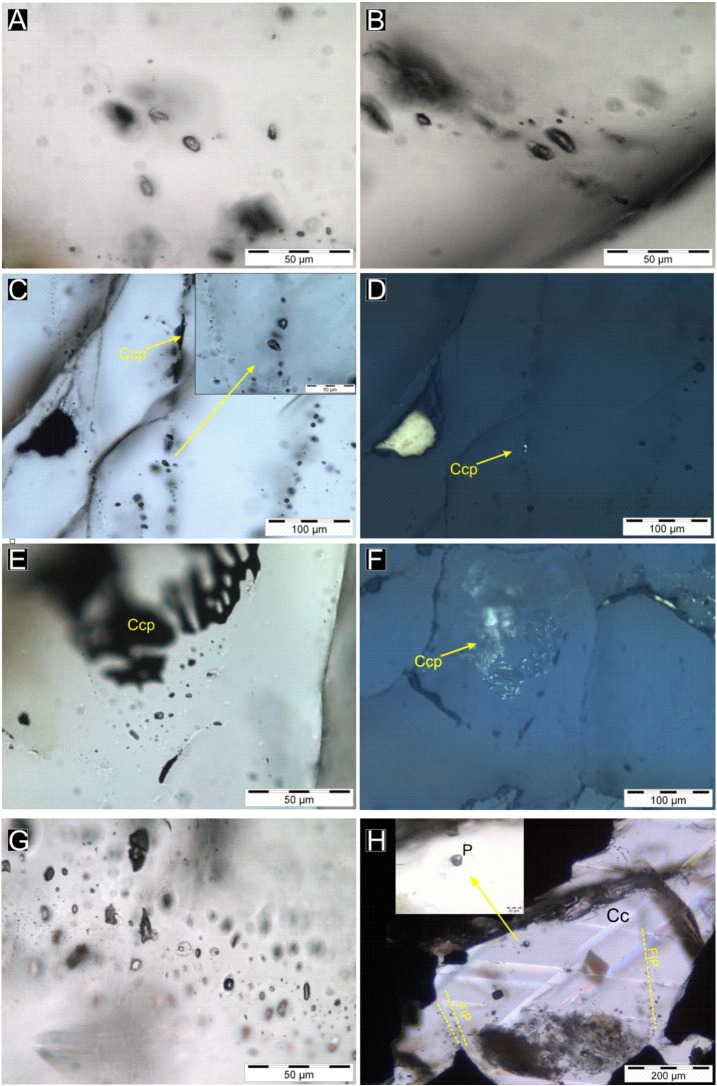
(A) Cluster of Type I, early CO_2_ fluid inclusions in the recrystallized quartz porphyroblast. (B) Three-phase (Laq, Lcar, Vcar) Type II, secondary CO_2_–H_2_O–NaCl fluid inclusions in the recrystallized quartz porphyroblast. (C) Three-phase Type II, secondary CO_2_–H_2_O–NaCl fluid inclusions in the recrystallized quartz porphyroblast. Note the chalcopyrite inclusions along the fluid inclusion planes. (D) Same area as panel C under reflected light. (E) Type III CH_4_–N_2_–H_2_O secondary fluid inclusions associated to chalcopyrite inclusions in fluid inclusion planes. (F) Same area as panel E under reflected light. (G) Pure CH_4_–N_2_ and H_2_O–NaCl inclusions along the same fluid inclusion trail. (H) Primary and secondary Type IV CaCl_2_–NaCl–H_2_O fluid inclusions in calcite II.

Type I Carbonic inclusions were observed in the quartz–porphyroblast, 10 m below the intrusion–footwall contact. The fluid inclusions occur isolated, or randomly in three-dimension arranged clusters (Figs. 6 and 9A). These petrographic feartures are not necessarily evident for a primary origin (Goldstein, 2003); however, the fact that these inclusions were never observed along healed microfractures supports a primary origin that is synchronous with the recrystallization of the host quartz. The inclusions are about 20–50 μm in size. Some inclusions have negative crystal shapes. At room temperature the inclusions contain two phases, a carbonic vapor (Vcar) and a carbonic liquid (Lcar) phase. The presence of a thin layer of aqueous liquid wetting the inclusion walls was not observed due to the limitations of resolution of the optical microscope (0.5 μm). Volume fraction of Vcar, determined by image analysis using the AnalySis Program package and the tables of Bakker and Diamond (2006), vary from 10 vol.% to 15 vol.%.
Type II These types of inclusions belonging to the CO_2_–H_2_O–NaCl system with highly variable phase ratios are arranged along fluid inclusion planes (FIPs) in the recrystallized quartz porphyroblast. At room temperature, the inclusions have three phases, with highly variable volume fractions; carbonic vapor (Vcar), carbonic-liquid (Lc) and aqueous-liquid (Laq) (Figs. 6 and 9B–C). Optically, the vast majority of the inclusions are two phase (Lcar–Vcar) with undetectable Laq phase. The presence of aqueous liquid was detected by Raman spectroscopy. In the aqueous-rich end-member, the volume fraction of the carbonic phase (degree of filling = DF) is around 5 vol.%. In inclusions with intermediate volume fractions, three phases (double bubble) were observed. In these inclusions, the volume fraction of the carbonic phase (Laq/Lcar + Vcar) is highly variable, but the volume fraction of the Vcar at room temperature is a constant ~ 30 vol.%. In some trails of the Type II FIA, chalcopyrite solid inclusions are observed (Figs. 6 and 9C–D).
Type III These types are characterized by the CH_4_–N_2_–H_2_O type composition, and these inclusions occur along fluid inclusion planes in the recrystallized quartz porphyroblast and in apatite, which host the actinolitic alteration zones. They define one phase vapor or two phase liquid + vapor inclusions (Figs. 6 and 9E–G). At room temperature, the majority of the inclusions are apparently dark, one phase vapor (Vm) inclusion, with variable sizes from 5 μm up to 50 μm. The presence of a liquid phase (Laq) is observed only if the volume fraction of the Vm ratio is less than 0.8 vol.%. Inclusions with intermediate Vm/Laq ratios (0.8–0.2) are very rare. In the liquid rich end-member, the volume fraction of the Vm ratio is around 20 vol.% (Fig. 9G). Chalcopyrite inclusions along the trails of Type III FIA are common and a chalcopyrite grain as solid inclusion in a large fluid inclusion is also observed (Figs. 6 and 9E–F).
Type IV These types of FIA are characterized by the H_2_O–NaCl–CaCl_2_ system and they appear: (1) as primary and secondary inclusions in calcite II that is intergrown with prehnite and pumpellyite in the hydrothermal veins (Fig. 9H); (2) as secondary inclusions along FIPs in the rock forming quartz crystals of the GRB, in 11 kilometer distance from the intrusion–footwall contact at Ely (Fig. 1C); and (3) as secondary inclusions in FIPs in the recrystallized quartz porphyroblast. Volume fraction of vapor is 10 vol.% in the calcite II and partly in the recrystallized quartz porphyroblast. Volume fractions of aqueous vapor in quartz of the GRB and partly in the recrystallized quartz porphyroblast are around 20 vol.%. The size of the inclusions ranges from 10 μm to 40 μm.

#### 4.4.2 Fluid inclusion microthermometry and Raman spectroscopy

Results of fluid inclusion microthermometry and the calculated salinities are summarized in Table 3.

**Fig. 10:**
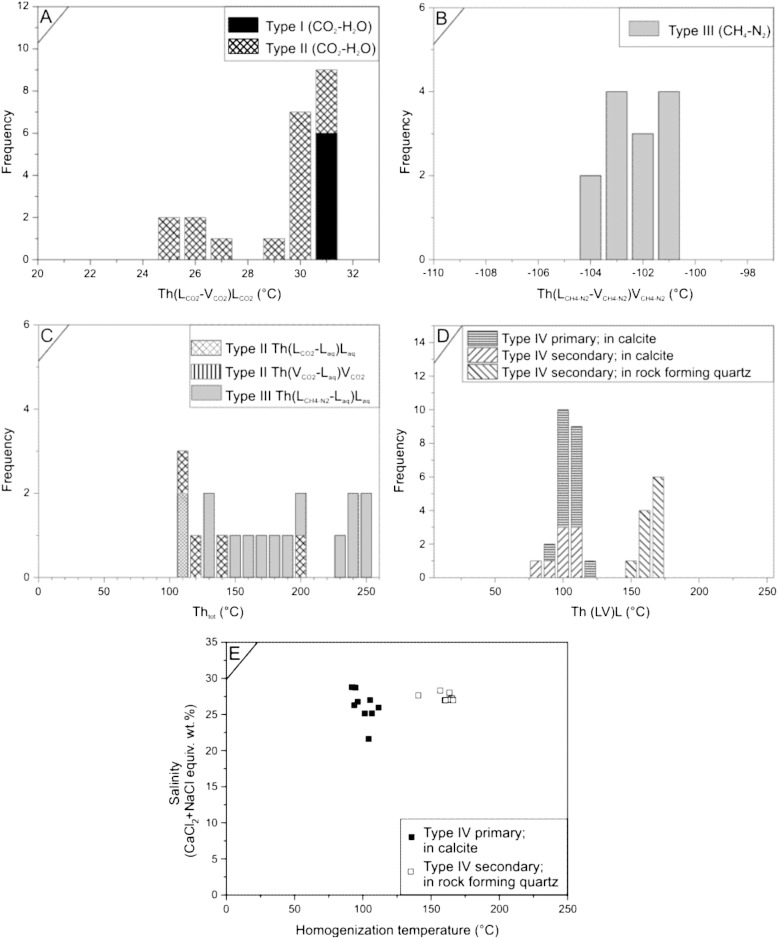
Microthermometric properties of Type I–IV fluid inclusion assemblages. (A) Homogenization temperature distribution diagram of the carbonic phase of Type I and II fluid inclusion assemblages. (B) Homogenization temperature distribution of CH_4_–N_2_ bearing fluid inclusions (Type III fluid inclusion assemblage). (C) Homogenization temperatures of the aqueous inclusions associated to the Type II and III fluid inclusion assemblage. (D) Homogenization temperatures of Type IV Ca-bearing aqueous fluid inclusions. (E) Homogenization temperature–total salinity diagram of the Type IV fluid inclusion assemblage.

The *Type I FIA* melting temperatures of CO_2_ is close to − 56.6 °C (− 56.7 °C to − 56.6 °C) indicating pure CO_2_ chemistry. The inclusions homogenize into the liquid phase between 30.7 °C and 31.0 °C (Fig. 10A). The bulk molar volume (ca. 79.43–85.82 cm^3^/mol) and the density range (0.51–0.55 g/cm^3^) for the inclusions were calculated from the homogenization temperatures.

*Type II FIA* melting temperatures of the CO_2_ in the Lcar-rich inclusions is − 56.6 °C, indicating no other volatiles (CH_4_ or N_2_) than CO_2_ are in the inclusions. Observation of eutectic melting around ~ 21 °C was possible only in one inclusion due to the small size of the aqueous inclusions or due to the low H_2_O/CO_2_ ratio. Ice melting and clathrate melting temperatures varied from − 3.9 °C to − 3.2 °C and from 8.4 to 8.9 °C, respectively. Salinities calculated from the clathrate melting temperatures vary from 1.8 NaCl equiv. wt.% to 2.7 NaCl equiv. wt.%. Homogenization temperatures of the carbonic phase into Lcar range from 24.7 °C to 31 °C (Fig. 10A). Molar volumes and densities range from 60 to 224 cm^3^/mol and 0.71 to 0.19 g/cm^3^, respectively. Total homogenization temperatures with homogenization into Laq vary from 105 °C to 192 °C. Bulk molar volume and density of the aqueous-rich end member are 19.35 cm^3^/mol and 0.98 g/cm^3^, respectively. The presence of carbonic vapor in the aqueous-rich end member was checked by Raman spectroscopy. Homogenization of the carbonic-rich end member into Vcar is difficult due to the optical limitations (Diamond, 2001), and is, therefore, the sequential heating method of Dubessy et al. (1992) and Berkesi et al. (2009) has been applied to measure total homogenization in the gas-rich inclusions. Analysis of two inclusions resulted in homogenization temperatures of around 105 ± 5 °C that equals the homogenization temperatures measured on aqueous-rich inclusions (Fig. 7C).

*Type III FIA* liquid phase in these inclusions appears only after significant undercooling (~ 160 °C), and the homogenization temperature of the Lm and Vm into Vm phase varies between − 121 °C and − 101.4 °C (Fig. 10B). These temperatures fall between the critical points of the pure CH_4_ (− 82.6 °C) and pure N_2_ (− 147.0 °C) system. According to the analysis of the Raman spectra, using the method of Burke (2001), the CH_4_/N_2_ ratio shows large variation between 0.15 and 1 with an average of 0.3. Molar volume and density for the gas-rich end member vary from 112.9 cm^3^/mol to 119.6 cm^3^/mol and from 0.21 g/cm^3^ to 0.23 g/cm^3^, respectively. Melting temperatures of ice phase in the aqueous-rich end members vary between − 0.7 °C and − 0.1 °C. Salinities vary between 0.23 and 1.25 NaCl equiv. wt.%, assuming an average CH_4_/N_2_ ratio of 0.15 and an average homogenization temperature of − 103 °C. Bulk molar volumes for aqueous end members vary from 19.51 mol/cm^3^ to 19.55 mol/cm^3^ and the densities range from 0.92 g/cm^3^ to 0.931 g/cm^3^. Homogenization temperatures of the aqueous end members vary between 126 °C and 251 °C (Fig. 10C) and the inclusions homogenized always with the disappearance of the vapor into liquid phase.

*Type IV FIA* eutectic temperatures (from − 52.3 °C to − 48.6 °C) were detected close to the eutectic temperature (~− 52 °C) for the H_2_O–NaCl–CaCl_2_ system. Melting of the hydrohalite and ice phases were observed from − 36.3 °C to − 19.4 °C and from − 27.8 °C to − 12.3 °C, respectively. Salinities range from 21.6 to 28.8 NaCl + CaCl_2_ equiv. wt. % are calculated from the hydrohalite and ice melting temperatures (Fig. 10D). The CaCl_2_/(CaCl_2_ + NaCl) ratio range between 0.3 and 0.6. Homogenization of the inclusions occurred with the disappearance of the vapor phase (L + V → L) between 84.5 and 166.2 °C. In the uncontaminated rock forming quartz of the footwall granite and prehnite–pumpellyite–quartz–calcite alteration zones in the WM-002 drill core, homogenization temperatures range from 160 °C to 170 °C and between 110 °C and 120 °C, respectively (Fig. 10D–E).

## 5 Discussion

### 5.1 Pressure and temperature conditions of fluid flow

#### 5.1.1 Early Type I CO_2_-rich fluids related to the peak metamorphic stage

During the contact metamorphism of the granitic footwall rocks within 10 m of the contact of the SKI at the Spruce Road area, almost all primary rock forming minerals were intensively resorbed, partially melted (Sawyer, 2002). During partial melting, quartz is one of the first reactant minerals, and therefore, the presence of primary magmatic quartz and formation of a single large quartz porphyroblast just 10 m beneath the contact is rather ambiguous. Since it is surrounded by a millimeter thick pyroxen + sulfide (chalcopyrite + pyrrhotite) halo that is interpreted as a peritectic partial melt product of pre-metamorphic epidote–actinolite veins (Benkó et al., 2013) one could argue the quartz is a partial melt product. However, the fact that (1) partial melt is composed of feldspars + quartz + biotite in eutectic composition, (2) the unique occurrence of the large quartz crystal and (3) and the enveloping texture of the pyroxene suggest that the quartz was preserved during the metamorphism and that metamorphism resulted in the recrystallization of the quartz porphyroblast.

Peak metamorphic temperatures calculated for contact metamorphic conditions, based on two-pyroxene thermometry (820–920 °C, Benkó unpublished data; > 800 °C, Sawyer, 2002), were used to carry out the pressure correction of the Type I FIA. Isochors were calculated for bulk molar volumes (Vm) of 79.4 cm^3^/mol and 85.82 cm^3^/mol (calculated from Th_car_ = 30.7 °C and 31.0 °C).

**Fig. 11:**
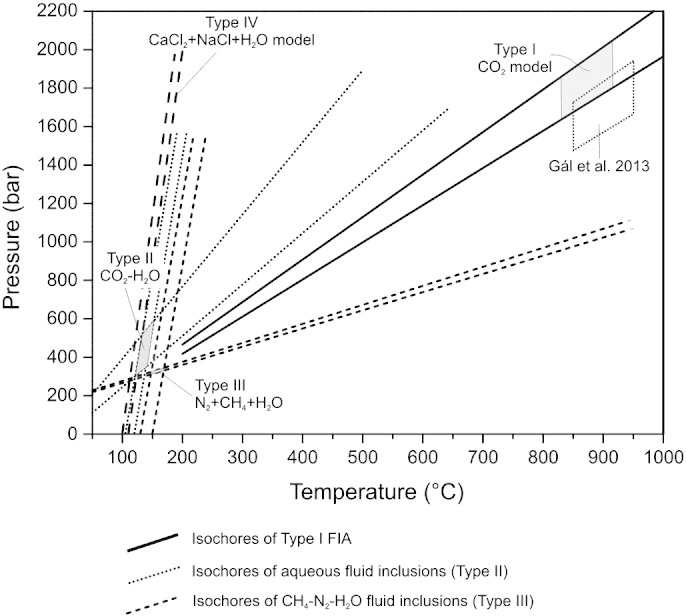
Trapping conditions of the Type I, II and III fluid inclusion assemblages. Isochores were calculated using equations from Zhang and Frantz (1987) for aqueous inclusions, and equations of Kerrick and Jacobs for the carbonic inclusions a in the FLINCOR program (Brown, 1989). Abb.: FIA—fluid inclusion assemblage.

The isochors intersect the 830 °C and 920 °C temperatures between 1.61 and 2.02 kbar (Fig. 11). This pressure correspond to the p–T conditions calculated (1.68–1.73 kbar) by Gál et al. (2013) who calculated for partial melt veins in the basal mineralized zone of the SKI, and those estimated by Turner (1968; 1.5–2.5 kbar).

Based on the calculated pressures (1.68–1.73 kbar) the thickness of the overburden rock thickness can be calculated. Assuming lithostatic conditions (3000 kg/m^3^) the overburden during the peak metamorphism could be 5.4–6.7 km. This thickness is much higher than the current location of the sample below the surface (300 m), but less than the estimated total thickness of the DC (Layered series + Volcanic Rocks = 11 km; Fig. 1B). The thickness of the volcanic and intrusive rocks decreases drastically towards the rims of the MCR at the Duluth Complex. The thickness of the volcanic and sedimentary rocks in the center of the valley may rich 11 km whereas in the external zones it decreases locally to 0 km. Therefore, adopting the arguments of Gál et al. (2013) the studied segment of the drill core at the rim of the SKI may represent a transitional zone between the internal and external part of the rift zone.

#### 5.1.2 Mixing of aqueous and carbonic-rich fluids

In the fluid inclusion planes of the Type II FIA (carbonic–aqueous inclusions) and Type III (CH_4_–N_2_ inclusions), the coexistence of gas-rich and aqueous liquid-rich inclusions with various degree of filling indicate either: (1) heterogeneous entrapment in an immiscible fluid system, or (2) mixing of two fluids with different compositions (Diamond, 2001; Hurai, 2010; Ramboz et al., 1982). Based on the microthermometric studies and analysis of large number of fluid inclusions by Raman spectroscopy, no fluid inclusions were detected with intermediate compositions (CO_2_–CH_4_–N_2_–H_2_O). Therefore, mixing or partial mixing of the Type II and III FIA can be excluded. The two FIA, therefore, indicate two different fluid mixing events.

If we assume that the heterogeneous trapping of the Type II FIA is the result of the mixing of two originally homogeneous fluid systems, than the trapping temperature can be calculated by the intersection of the isochors of the aqueous-rich (3.4 NaCl equiv. wt.%, Th = 105–130 °C) and the carbonic-rich (Thcar = 24 to 31 °C) end members (Mullis, 1987; Roedder, 1984). The pressures assuming fluid mixing, varies from 240 to 650 bar and 120–150 °C (Fig. 11). These pressures under hydrostatic conditions correspond to 2400 to 6500 m depth, whereas under lithostatic conditions 800–2200 m.

The texture of the Type III (CH_4_–N_2_–H_2_O) inclusions also displays heterogeneous trapping. Similar to the Type II assemblage, mixing of two primarily homogeneous fluid phases is assumed. The representative isochors of gas-rich inclusions are calculated for molar volumes of 113 and 120 mol/cm^3^, and those of the aqueous-rich inclusions for Th = 130 °C and 150 °C. The isochors' intercept is in the range between 315 and 360 bar and 145 °C to 165 °C (Fig. 11). Under hydrostatic conditions, these pressures are equivalent with 3150 to 3600 m.

The calculated pressures, temperatures, and depth intervals do not correspond to depth estimations that are calculated from the Type I primary inclusions (5.4–6.7 km).

To explain these discrepancy in the pressure and temperature conditions, three scenarios are proposed: (1) selective diffusion of volatiles through the fluid inclusion wall after entrapment that changes the primary bulk composition (i.e., slope of isochors), or (2) diagenetic origin of fluids. The first process has been proposed by Bakker and Jansen (1994) and Doppler and Bakker (2012). They observed that at even in unstrained quartz crystals, with increasing temperature, the inclusions easily lose D_2_O (deuterium-oxide) and re-equilibrate. Hydrogen diffusion, as it was suggested by Hall et al. (1991) and Morgan et al. (1993), may result in the formation of methane in CO_2_ inclusions or precipitation of graphite and hydrogen in methane-bearing inclusions. Loss of water from carbonic inclusions may also trigger precipitation of graphite. Neither graphite or hydrogen in the inclusions or inclusions with variable CO_2_/CH_4_ ratios were detected, therefore, hydrogen diffusion or selective water leakage likely did not change the bulk composition of the inclusions.

According to the second scenario, the metasedimentary Virginia Formation or the hornfelsed Virginia Formation inclusions in the SKI may have released carbonic-rich fluids due to the groundwater–rock interaction any time after the formation of the SKI. These fluids may have migrated in the SKI and in the neighboring rock volumes and along some preferred permeable zones. This latter model also explains why no volatile-rich fluids were encountered far (~ 10 km) from the contact in the GRB.

### 5.2 Formation conditions of hydrothermal veins

The actinolite + cummingtonite + chlorite + albite + quartz + calcite + prehnite + pumpellyite (hydrothermal veins hereinafter) assemblage corresponds to the characteristic mineral assemblages of lower greenschist–prehnite–pumpellyite facies (> 250 °C at 2 kbar; Best, 2012).

To estimate temperatures in the hydrothermal veins, the chlorite thermometry is applied. Compositions of the chlorite in the studied hydrothermal veins are comparable with those reported from the Layered and Anorthositic Series of the DC (South Filson Creek area, SKI; Gál et al., 2011) and volcanogenic massive sulfide deposits (Phelps Dodge deposit; Kranidiotis and MacLean, 1987), but differ markedly from those reported from Archean gold deposits (Zang and Fyfe, 1995) or from active geothermal systems (Salton Sea; Cathelineau, 1988; Fig. 7B and C).

A systematic relationship between the formation temperature of chlorite and the Al^IV^ (aluminum in the tetrahedral position) in the crystal structure was first reported by Cathelineau (1988). The fundamental equation for temperature estimation of Cathelineau (1988) has been modified several times for different rock environments by Kranidiotis and MacLean (1987) and Zang and Fyfe (1995). These equations also consider the relationship between temperature and Fe in the octahedral position. Comparison of compositions of chlorites from the footwall and the SKI and the chlorite analyses of Cathelineau (1988); Kranidiotis and MacLean (1987) and Zang and Fyfe (1995) are plotted on Fig. 7B and C. The Al^IV^ vs. Fe/(Fe + Mg) and the Si/Al vs. Fe/(Fe + Mg) ratios (both given in *apfu*) show that the analyzed samples are comparable with the composition fields of Kranidiotis and MacLean (1987). Therefore, for further temperature calculation, only temperature data calculated with their equations are applied. Chlorite formation temperatures (Table 1) vary according to Kranidiotis and MacLean (1987) between 276 °C and 308 °C (Fig. 4D). The stability field of prehnite may extend as high as 400 °C, but characteristically, it forms around 220 °C in hydrothermal systems (Reyes, 1990; Wheeler et al., 2001) that is close to the temperatures calculated from composition of chlorite.

The Type IV FIA does not show any evidence of heterogeneous trapping. Therefore the homogenization temperatures (110–120 °C and 160–170 °C) represent the minimum temperature of trapping. In order to obtain the trapping pressures, an independent thermometer, or barometer is required. A possible candidate would be the prehnite with which the calcite II is apparently syngenetic, but prehnite precipitates only if the activity of the CO_2_ is lower than 0.01 in the system. Even if the isochors of these inclusions are intercepted with the 1.7 kbar isobar (trapping pressure of Type I primary fluid inclusions), the trapping temperatures do not exceed the 200 °C. Therefore, calcite II can be considered as the last phase to precipitate during hydrothermal fluid flow.

### 5.3 Origin of hydrothermal fluids

#### 5.3.1 Origin of carbonic fluids

Pure or nearly pure CO_2_-bearing inclusions are common in charnockites (Banerjee et al., 2013). Pressure and temperature conditions of trapping, calculated for carbonic inclusions, are generally consistent with the P and T conditions of the host rock; therefore these inclusions are believed to have formed close to the peak of the metamorphism (Hollister, 1988). Since almost pure CO_2_ inclusions are very rare in the nature, the nearly pure CO_2_ composition, the origin and the transport of the carbonic phase require explanation.

Carbonic phases in migmatites generally originated from: (1) mantle degassing, (2) decarbonation of carbonate-bearing lithologies (Glassley, 1983; Holloway, 1976), or (3) reaction of graphite, aqueous fluids and hydrous minerals (Hollister and Burruss, 1976; Lamb and Valley, 1984). Only the Early Proterozoic Virginia Formation contains graphite and carbonate beds. Decarbonation of these metasedimentary rocks, upward migration of carbonic fluids, as well as, strong textural relationship of graphite and Cu–Ni–PGE sulfide mineralization in the basal mineralized zone of the layered intrusions has been widely reported in the past decades (Mogessie and Stumpfl, 1992; Mogessie et al., 1991; Pasteris et al., 1995; Ripley, 1990). However, these rock units are not found in the Spruce Road deposit, since this rock units were probably digested by the intruding troctolitic magma. Evidence for the presence of the metasedimentary rocks prior to the emplacement of the SKI magma are the hornfels inclusions in the basal zone of the SKI (Patelke, 2003). The chalcopyrite–pyrrhotite-rich sulfide melt in the pyroxene veins is derived from the basal mineralized zone therefore it is proposed that the carbonic-rich fluids may have also migrated along these partially molten shear zones from the graphite-bearing Virginia Formation-rich basal mineralized zones into the charnockitic footwall.

Recently, Banerjee et al. (2013) modeled CO_2_ migration in charnockitic rocks. They found that percolation of CO_2_ along grain boundaries is inhibited due to the large wetting angles but possible and fast (338 mm in 500 years) along microfractures. CO_2_ diffusion in melts, however, is only possible above ~ 840 °C. Considering that the temperature in the proximal 10 m part of the footwall of the DC was between 830 and 910 °C and the granite was in a partially molten state, both processes could play role in the CO_2_ diffusion into the granite, but possibly the presence of high permeable zones (pyroxene-rich veins) promoted the CO_2_ diffusion.

#### 5.3.2 Origin of calcium-bearing fluids

Calcium-bearing, high salinity fluids (Type IV) have been found as primary inclusions in calcite II in the hydrothermal vein assemblage and in the rock forming quartz of the GRB 10 km away from the intrusion–footwall contact. Ca-rich high salinity fluids are commonly found in sedimentary basins and on shields where the high salinity is the result of the significant fluid–rock interaction under low fluid/rock ratio conditions. Therefore, these fluids are considered as regional formational fluids. Groundwater in the Soudan Mine (~ 30 km away from the Duluth Complex) has been recently investigated by Holland et al. (2013) and Alexander pers. comm. (2013). They reported that the composition (Ca-rich, ~ 25 NaCl + CaCl_2_ wt.%) of regional formational fluids do not show any significant variation in the past 2 Ga years. The reported compositions are comparable with the compositions of the fluid inclusions discussed in this work.

### 5.4 Origin of sulfur

Sulfur isotope compositions (δ^34^S) of sulfide assemblages related to the partial melting have average values of approximately + 8‰ in the footwall, and are comparable with the sulfide isotope ratios in the basal zones of the intrusion (this study). The similar values indicate that during the infiltration of the sulfide liquid into the footwall, along some permeable zones created by the partial melting (Hovis, 2003; Sawyer, 2002), there was essentially no isotopic fractionation between the sulfide liquid and the high temperature sulfide minerals. However, the comparable sulfide isotope values in the footwall and in the basal mineralized zone is direct evidence that the sulfide most likely was derived from the intrusion.

At high temperatures (~ 800 °C) isotopic fractionation is minor and hence can be neglected (e.g., Ohmoto and Rye, 1979). However, with decreasing temperature, the significance of isotopic fractionation increases, although the magnitude of the isotope fractionation depends upon the fluid species and minerals involved (Ohmoto and Rye, 1979). In addition, isotopic fractionation depends not only on temperature, but also on the pH, *f*O_2_, *f*S_2_ and the total S content of the fluid (Ohmoto, 1972; Ohmoto and Rye, 1979). According to Sakai (1968) and Ohmoto (1972), in a hydrothermal fluid SO_4_^2−^ strongly sequesters ^34^S relative to sulfide. Therefore, sulfide minerals precipitating from a relatively oxidizing hydrothermal fluid will have lighter δ^34^S values relative to the total dissolved sulfur in the hydrothermal fluid (Ohmoto, 1972; Ohmoto, 1986; Ohmoto and Rye, 1979). We propose, based on the petrographic evidence, that the hydrothermal fluids dissolved sulfide minerals with sulfur isotope values around + 8‰. During the re-precipitation from low-temperature hydrothermal fluid under high oxygen fugacity conditions, due to the isotopic fractionation indicated above — the precipitated sulfide minerals will have lighter δ^34^S values than the dissolved sulfur in the hydrothermal fluid. δ^34^S values around + 5.4 to + 5.7‰ validate this relationship between sulfides with magmatic and hydrothermal origin.

Under certain conditions, variation in pH may also cause variation in sulfur isotope ratio. Chalcopyrite in association with calcite II indicates increasing pH. Increasing pH will shift δ^34^S of HS^−^ and H_2_S (aq) to lighter values vs. total dissolved sulfur, and hence δ^34^S value of sulfide minerals in the hydrothermal veins will decrease vs. sulfur in the hydrothermal fluid (Ohmoto, 1972; Ohmoto and Rye, 1979). This process may also have contributed to the decrease in sulfide isotope values in the hydrothermal veins.

To sum up, either the increasing or high oxygen fugacity or/and the increase of pH lead to the precipitation of hydrothermal chalcopyrite having lighter sulfur isotope values relative to the primary sulfide assemblage (Fig. 8).

### 5.5 Fluid–rock interaction and base metal redistribution

Chalcopyrite inclusions in the Type II and III fluid inclusion assemblages indicate that these fluids played a role in metal redistribution. However, these solid inclusions are found associated with CO_2_ (Type II) and CH_4_ (Type III) bearing fluids only in the recrystallized quartz porphyroblast, which is mantled by chalcopyrite and pyrrhotite. Therefore, the estimation of the extent of this remobilization is difficult.

Hydrothermal veins with comparable mineral assemblages have been reported from the Layered and Anorthositic Series of the Partridge River intrusion and SKI (Gál et al., 2011, 2013; Mogessie and Saini-Eidukat, 1992; Mogessie et al., 1991). These studies suggested that one type of hydrothermal fluids was probably Ca-bearing, high salinity fluid, based on the calcic alteration around the chlorite veins in plagioclase feldspars. Based on fluid inclusion studies, our work confirms this model. Temperature estimation for chlorites in the hydrothermal veins (from 250 to 350 °C) of Gál et al. (2011) overlap perfectly with our calculations. Gál et al. (2011) also proved that the hydrothermal fluid flow has been controlled by some major fault zones related to the rifting. A clear, fault related occurrence of hydrothermal mineralization was not confirmed in the footwall, but the mineralization is characteristically associated to partial melt veins, that are probably associated to the tectonic activity during the rift formation. Based on the mineralogical, geochemical similarities and on the overlap in temperature estimations, we conclude that the hydrothermal formations in the footwall and within the DC may belong to the same, large-scale hydrothermal fluid flow system. Mogessie et al. (1991) and Mogessie and Saini-Eidukat (1992) reported PGE mineralization associated to the chlorite-bearing hydrothermal veins, whereas similar association was not confirmed by Gál et al. (2011). PGM in association with hydrothermal veins have not been found in the footwall during this study. Notwithstanding, it has to be noted that this might account for the PGE-poor character of the remobilized ore.

Hydrothermal alteration assemblages and Ca-bearing fluids have been found in the SKI, Partridge River intrusion and Bathtub intrusion (Benkó et al., 2013) of the Duluth Complex, as well as, in the granitic footwall at the Spruce Road deposit. Schmidt (1993) reported regional, low-grade burial metamorphism of the Keweenawan basalts (North Shore Volcanics; Fig. 1B) characterized by albite, epidote, prehnie, actinolite, chlorite and albite. Jolly and Smith (1972) and Jolly (1974) presented evidences, that fluids associated to this low-grade burial metamorphism were able to remobilize Cu and Zn from the volcanic rocks. These evidences raise the question if the alteration in the footwall of the SKI is necessarily associated to the formation of the MCR. Considering, that the composition of shield fluids did not change considerably in the past 2 Ga (Holland et al., 2013), the alteration may have happened any time after the formation of the Duluth Complex.

## 6 Conclusion

Four different fluid inclusion assemblages have been distinguished in the charnockitic footwall of the SKI at the Spruce Road deposit. Apparently pure CO_2_-bearing fluid inclusions were found as primary inclusions in a recrystallized quartz porphyroblast with near critical homogenization temperatures (30.7–31 °C). Assuming that these inclusions trapped during the peak metamorphism when the temperatures were in the range from 830 to 920 °C, the calculated pressure of trapping varies between 1.6 and 2.0 kbar. This pressure range corresponds to earlier pressure estimations, carried out in the felsic dykes at the bottom of the SKI (Gál et al., 2013). The CO_2_ in the inclusions can be derived from the Paleoproterozoic graphite bearing Virginia Formation, found as inclusions in the basal zones of the SKI, which was decarbonated during the contact metamorphism. A possible explanation for the apparently pure CO_2_ composition of the inclusions is that the H_2_O that percolated primarily with the CO_2_ in the system reacted with the silicate minerals forming secondary hydrous silicates.

Fluid inclusion assemblages with CO_2_ + H_2_O and CH_4_ + N_2_ + H_2_O compositions migrated after the peak of the contact metamorphism along some permeable zones in the charnockite. Based on petrographic evidence for both assemblages, the mixing of a low salinity aqueous and a low density carbonic fluid can be modeled. Trapping temperatures and pressures were calculated using the isochore interception method of the pure end-members. The obtained pressures range between 240 to 650 bar and 315 to 360 bar, respectively. These pressures are far lower than those calculated for the primary inclusions. Therefore these fluids, trapped probably any time after the formation of the SKI during the exhumation of the DC. The carbonic phase can be derived from the decarbonation of the graphite-bearing metasedimentary Virginia Formation inclusions. Both fluid phases played a significant role, at least at local scale in the remobilization and redistribution of copper along some fracture zones in the charnockite. In order to estimate the economic significance of hydrothermal remobilization a more extended work is needed involving several drill core samples and a detailed geochemical work is needed to evaluate the role of fluids in PGE redistribution

Ca-bearing, high salinity fluids resulted in formation of pervasive or vein type lower greenschist to prehnite–pumpellyite facies alteration. In these zones the actinolite + cummingtonite → chlorite + calcite I + quartz + albite + magnetite → prehnite + pumpellyite + calcite II + chalcopyrite indicate cooling from high (> 250–300 °C) to low temperatures (< 200 °C). Composition of fluid inclusions (Ca-bearing, high-salinity) found in the second generation of calcite are comparable with current groundwater compositions and hence involvement of shield brines in the late stage fluid–rock interaction related to the cooling of the contact aureole of the Duluth Complex. Since Ca-bearing high-salinity fluids resulted in alterations with similar alteration assemblages in several units of the Duluth Complex, the possibility of a post-Keweenawan fluid flow event cannot be excluded.

Sulfur isotope studies have revealed that the different sulfide assemblages characterized by sulfur isotope values (δ^34^S ≈ 8‰) infiltrated primarily during the contact metamorphic process from the intrusion into the footwall. During the hydrothermal remobilization due to the preferred fractionation of ^32^S in the sulfide phase, the δ^34^S values will decrease compared to the source sulfur isotope values. Accordingly, the measured low δ^34^S values range around + 5.5‰.

## Figures and Tables

**Table 1:** Representative composition of chlorites, used for chlorite thermometry.

Drill hole	WM-002	WM-002	WM-002	WM-002	WM-002	WM-002	WM-002	WM-002	WM-002	WM-002
Depth (m)	206	206	206	206	196.6	196.6	196.6	196.6	196.6	196.6
Petrographic position	Chlorite intergrown with quartz and calcite									
Sample no.	Z2-41	Z2-41	Z2-41	Z2-41	Z2-29	Z2-29	Z2-29	Z2-29	Z2-29	Z2-29
Mineral	Clin	Clin	Clin	Clin	Clin	Clin	Clin	Clin	Clin	Clin
*Reformatted oxide percentages based on 28 oxygens (with Fe^2 +^/Fe^3 +^ and OH calculated assuming full site occupancy)*										
SiO_2_	28.88	28.37	29.45	28.82	26.14	26.22	26.46	26.31	26.23	26.77
TiO_2_	0.00	0.08	0.01	0.14	0.10	0.07	0.09	0.06	0.16	0.13
Al_2_O_3_	19.78	19.48	19.60	19.43	18.62	18.68	18.67	18.72	18.78	18.54
Fe_2_O_3_	0.32	0.00	0.43	0.34	0.00	0.11	0.23	0.00	0.29	0.31
FeO	15.50	15.05	16.78	16.62	26.89	26.43	25.68	26.54	26.43	26.89
MnO	0.44	0.39	0.47	0.40	0.32	0.38	0.35	0.28	0.37	0.35
MgO	23.25	23.70	22.90	22.55	14.79	14.74	15.15	15.21	14.48	14.70
CaO	0.00	0.00	0.02	0.06	0.02	0.01	0.04	0.05	0.04	0.02
Na_2_O	0.00	0.00	0.00	0.00	0.00	0.00	0.00	0.02	0.00	0.00
K_2_O	0.02	0.12	0.01	0.01	0.04	0.04	0.04	0.00	0.00	0.00
H_2_O	12.08	11.96	12.18	12.03	11.15	11.14	11.19	11.22	11.15	11.26
Total	100.27	99.16	101.91	100.39	98.07	97.83	97.91	98.42	97.93	98.98

*Cations based on 28 oxygens*										
Si	5.73	5.68	5.78	5.74	5.62	5.64	5.66	5.62	5.64	5.70
Ti	0.00	0.01	0.00	0.02	0.02	0.01	0.01	0.01	0.03	0.02
Al	4.63	4.60	4.54	4.57	4.72	4.74	4.72	4.72	4.76	4.66
Fe^3 +^	0.05	0.00	0.06	0.05	0.00	0.02	0.04	0.00	0.05	0.05
Fe^2 +^	2.57	2.53	2.75	2.77	4.84	4.75	4.60	4.76	4.75	4.79
Mn	0.07	0.07	0.08	0.07	0.06	0.07	0.06	0.05	0.07	0.06
Mg	6.88	7.08	6.69	6.70	4.74	4.73	4.83	4.84	4.64	4.66
Ca	0.00	0.00	0.00	0.01	0.00	0.00	0.01	0.01	0.01	0.00
Na	0.00	0.00	0.00	0.00	0.00	0.00	0.00	0.02	0.00	0.00
K	0.01	0.06	0.01	0.01	0.02	0.02	0.02	0.00	0.00	0.00
OH	16.00	16.00	15.96	16.00	16.00	16.00	16.00	16.00	16.00	16.00
Total	39.55	39.58	39.71	39.74	41.86	41.75	41.59	41.78	41.73	41.76

*Parameters used for temperature calculation*										
Fe^2 +^ + Fe^3 +^	2.62	2.53	2.82	2.82	4.84	4.77	4.63	4.76	4.80	4.84
Si/Al	1.24	1.23	1.27	1.26	1.19	1.19	1.20	1.19	1.18	1.22
Al^IV^	2.27	2.32	2.22	2.26	2.38	2.36	2.34	2.38	2.36	2.30
Fe/(Fe + Mg)	0.28	0.26	0.30	0.30	0.51	0.50	0.49	0.50	0.51	0.51
T (°C) CAT, 1988	669	684	654	665	705	698	690	705	699	680
T (°C) K&M, 1987	279	283	276	279	308	305	302	307	306	300
T (°C) Z&F, 1995	265	271	258	261	255	253	252	256	253	246

Abbreviations: Clin = chlinochlore, CAT = Cathelineau, K&M = Kradidiotis and MacLean, Z&F = Zang and Fyfe.

**Table 2:** S isotope values in various ore assemblages at the Spruce Road area.

Sample no.	Depth (m)^a^	Host	Lithology	Mineral phase	Metamorphic/hydrothermal alteration assemblage	Sulfide texture	δ^34^S (‰ VCDT)	Reference^b^
Z2-31	20.4	Intrusion	Troctolite	Chalcopyrite + cubanite + pentlandite	–	Disseminated	8.25	Molnár et al. (2010)
Z2-6	120.1	Troctolite	Chalcopyrite	Chlorite + prehnite + calcite	Vein-type/disseminated	8.25
Z2-7	122.7	Troctolite	Chalcopyrite	Chlorite + prehnite + calcite	Vein-type/disseminated	8.90
Z2-14	151.8	Troctolite	Chalcopyrite + cubanite + pentlandite	–	Disseminated	7.81
Z2-16	163.1	Troctolite	Chalcopyrite + cubanite + pentlandite	–	Disseminated	7.38
Z2-17	168.6	Norite	Chalcopyrite + pyrrhotite + pentlandite	–	Massive	8.13
Z2-18	179.5	Norite	Chalcopyrite + pyrrhotite + pentlandite	–	Massive	8.86
Z2-23	187.1	Footwall	Charnockite	Pyrrhotite + pentlandite + chalcopyrite	–	Massive/net textured	9.04	Benkó et al. (2013)
Z2-46	226.5	Charnockite	Chalcopyrite	Chlorite + quartz + calcite + prehnite + pumpelleyite	Disseminated	5.46	this study
Z2-47	230.4	Charnockite/pyroxenite vein	Chalcopyrite + pyrrhotite + pentlandite	Pyroxenite vein	Massive, vein type	9.77	Benkó et al. (2013)
Z2-55	263.0	Charnockite	Chalcopyrite	Chlorite + quartz + calcite + prehnite + pumpelleyite	Disseminated	5.70	this study
Z2-54	257.9	Charnockite	Bornite–chalcopyrite	Quartz–biotite melt patches	Disseminated	8.61	Benkó et al. (2013)
Z2-58	272.8	Charnockite	Bornite–chalcopyrite	Quartz–biotite melt patches	Disseminated	8.11
Z2-63	300.2	Charnockite	Bornite–chalcopyrite	Quartz–biotite melt patches	Disseminated	7.57
Z2-68	306.6	Charnockite	Bornite–chalcopyrite	Quartz–biotite melt patches	Disseminated	8.76

a Distance from the top of the drill hole.

b Petrographic description of the host rock and the alteration assemblage.

**Table 3:** Microthermometric properties of the fluid inclusion assemblages.

Fluid inclusion assemblage	Composition	Habitus	Mode of entrapment	Host mineral	Volume fraction (%)		Tm_car_		Te	Tm_ice_		Tm_clathrate_		Th_tot_(L_car_V_car_) → L_car_		Th_tot_(L_car_L_aq_) → L_aq_	
Lcar + Vcar	Vcar	Min	Max	Min	Max	Min	Max	Min	Max	Min	Max
Type I	CO_2_–H_2_O	Early	Homogeneous	Quartz porphyroblast	99	10–15	− 56.6	− 56.6						30.7	31.0		
Type II	CO_2_–H_2_O–NaCl	Secondary	Heterogeneous	20–99	30	− 56.7	− 56.6	− 21.1	− 3.2	− 3.9	8.4	8.9	24.7	31	131	190


Fluid inclusion assemblage	Composition	Habitus	Mode of entrapment	Host mineral	Volume fraction (%)	Te	Tm_ice_		Th(L_m_V_m_) → V_m_		Th(L_aq_V_m_) → L_aq_	
Vm	Min	Max	Min	Max	Min	Max
Type III	CH_4_–N_2_–H_2_O	Secondary	Heterogeneous	Quartz porphyroblast	20–99	− 21	− 0.7	0.0	− 104.4	− 101.4	126	251.3


Fluid inclusion assemblage	Composition	Habitus	Mode of entrapment	Host mineral	Volume fraction (%)	Te		Tm_HH_		Tm_ice_		Na/Ca ratio		Total salinity		Th(LV) → L_(aq)_	
V	Min	Max	Min	Max	Min	Max	Min	Max	Min	Max	Min	Max
Type IV	H_2_O–NaCl–CaCl_2_	Primary and secondary	Homogeneous	Rock forming quartz in the GRB, calcite II	10	− 52.3	− 48.6	− 36.3	− 19.4	− 27.8	− 12	0.3	0.6	21.63	28.78	84.5	166.2
